# Bioelectric stimulation outperforms brain derived neurotrophic factor in promoting neuronal maturation

**DOI:** 10.1038/s41598-025-89330-4

**Published:** 2025-02-08

**Authors:** María del Pilar Diego-Santiago, María Ujué González, Esther María Zamora Sánchez, Nuria Cortes-Carrillo, Carlos Dotti, Francesc Xavier Guix, Sahba Mobini

**Affiliations:** 1 Instituto de Micro y Nanotecnología, IMN-CNM, CSIC (CEI UAM+CSIC), Madrid, Spain; 2 Molecular Neuropathology Unit, Physiological and Pathological Processes Program, Centro de Biología Molecular Severo Ochoa (CBM), CSIC-UAM, Madrid, Spain; 3 Grup d’Enginyeria de Materials (GEMAT), Institut Químic de Sarrià (IQS), Univeritat Ramon Llull (URL), Barcelona, Spain

**Keywords:** Neuronal differentiation and maturation, Electrical stimulation, Charge injection, Biochemical-free stimulation, Neural extracellular vesicles, Regeneration and repair in the nervous system, Electrochemistry, Biomedical engineering, Neuroscience

## Abstract

Neuronal differentiation and maturation are crucial for developing research models and therapeutic applications. Brain-derived neurotrophic factor (BDNF) is a widely used biochemical stimulus for promoting neuronal maturation. However, the broad effects of biochemical stimuli on multiple cellular functions limit their applicability in both in vitro models and clinical settings. Electrical stimulation (ES) offers a promising physical method to control cell fate and function, but it is hampered by lack of standard and optimised protocols. In this study, we demonstrate that ES outperforms BDNF in promoting neuronal maturation in human neuroblastoma SH-SY5Y. Additionally, we address the question regarding which ES parameters regulate biological responses. The neuronal differentiation and maturation of SH-SY5Y cells were tested under several pulsed ES regimes. We identified accumulated charge and effective electric field time as novel criteria for determining optimal ES regimes. ES parameters were obtained using electrochemical characterisation and equivalent circuit modelling. Our findings show that neuronal maturation in SH-SY5Y cells correlates with the amount of accumulated charge during ES. Higher charge accumulation (~ 50 mC/h) significantly promotes extensive neurite outgrowth and ramification, and enhances the expression of synaptophysin, yielding effects exceeding those of BDNF. In contrast, fewer charge injection to the culture (~ 0.1 mC/h) minimally induces maturation but significantly increases cell proliferation. Moreover, ES altered the concentration and protein cargo of secreted extracellular vesicles (EV). ES with large enough accumulated charge significantly enriched EV proteome associated with neural development and function. These results demonstrate that each ES regime induces distinct cellular responses. Increased accumulated charge facilitates the development of complex neuronal morphologies and axonal ramification, outperforming exogenous neurotrophic factors. Controlled ES methods are immediately applicable in creating mature neuronal cultures in vitro with minimal chemical intervention.

## Background

Neuronal differentiation is a critical process with wide-ranging applications in developmental biology^1^ regenerative medicine^2^, and cancer research^3^. For instance, neuronal differentiation is studied as a promising therapeutic strategy to suppress aggressive central nervous system cancers^4^. Furthermore, the induction of neuronal differentiation and maturation is a key objective for both the treatment of neurodegenerative diseases^5^, and the development of reliable in vitro models (test beds)^6,7^. In laboratory settings, neuronal differentiation and maturation are induced using retinoic acid (RA) followed by several doses of neurotrophic factors, such as brain-derived neurotrophic factor (BDNF)^8,9^. Upon entry into the cell, RA binds to its specific nuclear receptors and ultimately influences broad numbers of genes that regulate the cell cycle and arrest proliferation. RA also induces the expression of tyrosine kinase receptor B (TrKB), the specific high affinity receptor of BDNF. Exogenous or endogenous BDNF binds to TrKB, which further triggers molecular cascades promoting cell survival, neurite outgrowth, synapse formation, and the functional maturation of neurons^10–12^. Although biochemical protocols for neuronal differentiation and maturation are well established for several cell lines, including human neuroblastoma SH-SY5Y, the use of biochemical reagents as direct treatments is challenged by their complex delivery and concerns due to their extended effects in multiple cellular functions^13–15^. Therefore, creating a mature neuronal cell with minimal chemical intervention is highly desirable.

Recent advancements in electrobiology research have revealed the potential of low voltage electrical stimulation (ES) to guide cell fate and function without chemical intervention^16–18^. ES is a unique physical tool that is hypothesized to mediate the communication of specific messages with cells primarily via controlled biophysical changes at the cell membrane. An external electric field can be designed to replicate the natural endogenous electric field in the biological microenvironment, which is shown to be vital for neural tissues to thrive naturally^19–24^. ES also has been studied to promote regeneration and remodelling responses via mimicking and prolonging injury currents in damaged tissues, including bone^25^, skin^26^, and nerves^21,27,28^. Moreover, there are several studies suggesting that ES enhances neural stem cells survival and differentiation. This occurs primarily due to the increase of intracellular Ca^2+^ and the subsequent activation of pathways such as RAS-MAPK and PI3-Akt, which ultimately promote the expression of BDNF and other neurotrophic and regenerative factors^29–31^. However, ES is challenged by inconsistent and irreproducible results, due to insufficient knowledge on its mechanism and the lack of standard protocols. Indeed, it is not yet clear if there is a one-to-one relationship between ES parameters/regimes (e.g., amplitude, frequency, duty cycle, etc.) and specific biological responses.

Our primary objective in this work is to determine the extent to which ES contributes to neuronal maturation in the absence of exogenous neurotrophic factors. Moreover, we attempt to elucidate the meaningful role of ES parameters—specifically frequency and duty cycle—and explore whether the characteristics of the ES signal are crucial in conveying a targeted message to cells, in this case promoting neuronal maturation.

It is not possible to optimize and predict the effective ES paradigm only through blind empirical studies, as one can imagine infinite combinations of parameters and regimes. Rationalisation of the effective parameters and control of their interactions with extracellular space and cells are complex. The delivery of an electric signal to the cell culture gives rise to three main events: presence of electric field, charge injection, and electrochemical reactions. Factors such as mode of stimulation^16,17^, electrolyte and electrode characteristics, and signal characteristics (e.g., amplitude, shape, frequency, duty cycle) play important roles in determining the magnitude and characteristics of these events. Recently, a few research groups, including us, have emphasised the need to incorporate electrochemical studies into bioelectrical stimulation research to develop a comparable understanding of the physicochemical events that occur in the system upon ES^32–35^. The use of equivalent circuits^36^ and creation of *digital twins*^37^ are powerful tools for predicting the delivered electrical signal characteristics, which help design relevant ES protocols. We use this approach to define and analyse the ES regimes.

In this study, we investigate the potential of ES to bypass the use of neurotrophic factors to create mature neuronal cultures in vitro. We developed three voltage-controlled monophasic pulsed ES protocols, designed to operate within a safe voltage range to avoid harsh electrochemical reactions. For each ES protocol, we calculated the values for charge injected and accumulated and the effective field delivered. Subsequently, we explored how these ES regimes affect SH-SY5Y neuronal morphology, presynaptic marker expression, composition and concentration of secreted extracellular vesicles, as well as their possible influence on cell proliferation and cell cycle. We have observed that all protocols induce, to some extent, neuronal differentiation in the absence of BDNF. Moreover, the ES regime with the highest charge accumulation results in superior neuronal maturation compared to BDNF treatment.

## Results

### Analysis of electrical stimulation (ES) regimes

**Fig. 1:**
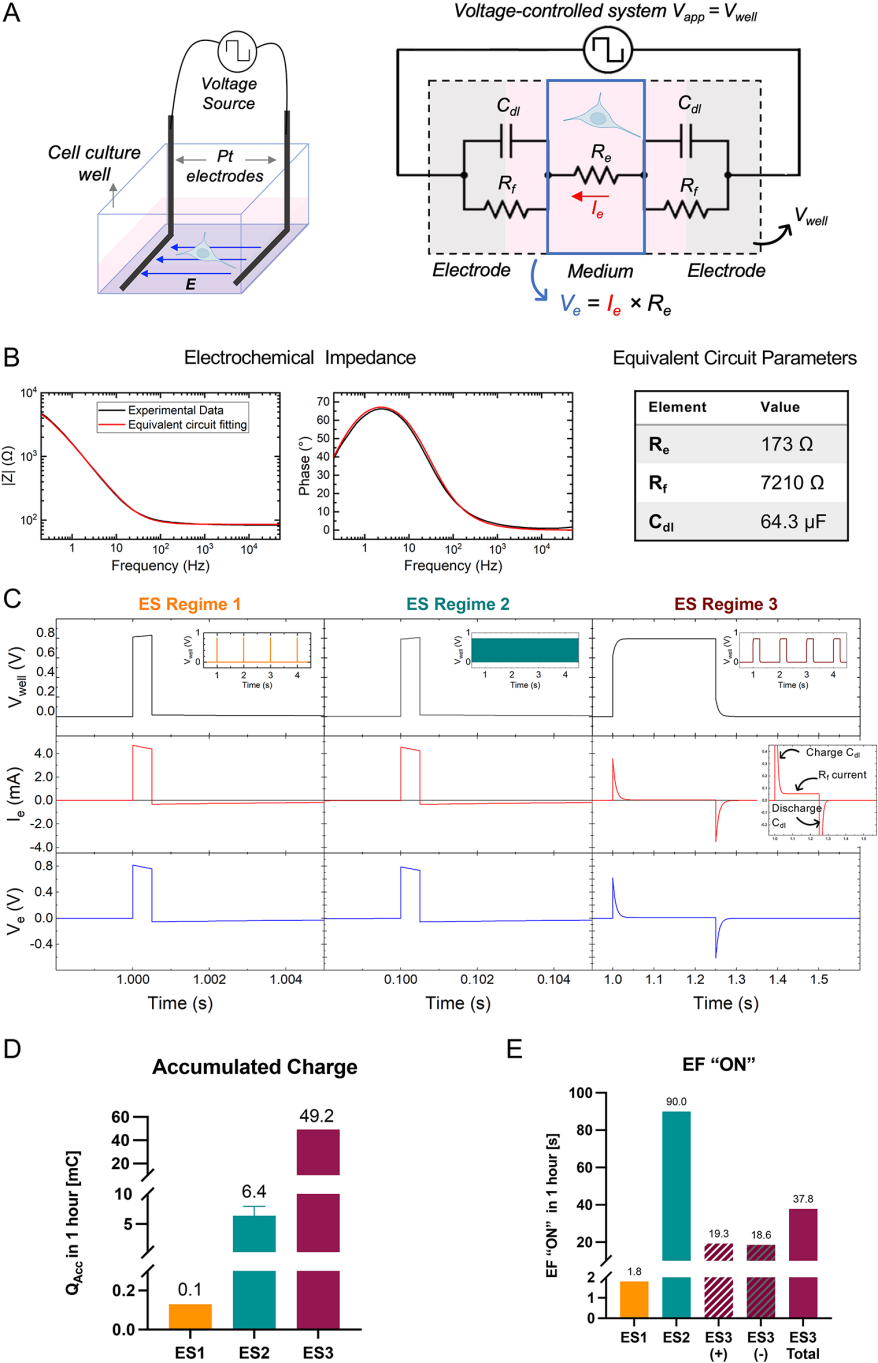
Analysis of electrical stimulation (ES) regimes. (**A**) Schematic of the direct ES device (left). Equivalent circuit, including elements representing the double layer capacitance (C_dl_), faradaic charge transfer resistance (R_f_), and electrolyte resistance (R_e_) (right). (**B**) Electrochemical impedance spectroscopy (EIS) of a single well measured experimentally (black), fitting to equivalent circuit (red), (left). The values of the components of the equivalent circuit resulting from the fitting appear in the table (right). (**C**) Applied voltage (V_well_), delivered current (I_e_), and voltage across the cell culture medium (V_e_) obtained from LT-Spice calculations using the equivalent circuit for each regime. (**D**) Accumulated charge (Q_Acc_) during one hour of stimulation for each ES regime. (**E**) Calculated effective electric field time (EF “ON”) for each ES regime. ES3(+) = EF “ON” during positive V_e_; ES3(−) = EF “ON” during negative V_e_; ES3 total = ES3(+) + ES3(−).

ES has been delivered to the cells by immersing Pt electrodes inside the cell culture medium (distance between electrodes, d = 32 mm)^31,38^, Fig. 1A. L-shaped Pt wires (0.6 mm in diameter) within the rectangular cell culture platform provide a homogeneous current distribution, as previously studied^39,40^.

The applied ES regimes are described in Table 1. Voltage-controlled monophasic pulsed ES has been chosen as a main mode of stimulation to control and keep the applied voltage within the safe window^41,42^. The safe window refers to the voltage range where water does not undergo electrolysis, and no harsh, irreversible electrochemical reactions are induced. Although electrochemical reactions are still taking place within the safe voltage range, they are effectively controlled and they are mostly reversible. The safe voltage window varies with environmental conditions, electrolyte composition, electrode configuration, and material and surface properties. This window was determined using cyclic voltammetry across different voltage ranges with DMEM as an electrolyte in biologically relevant conditions. We found that the safe window for our system is approximately [− 0.7 V, 1.1 V], where significant changes in the generated current do not occur (Supplementary Fig. S1). The voltage applied to the cell culture well (V_well_) has been fixed at 800 mV, which is a considerably large amplitude within this safe window. In our stimulation device, described in Fig. 1, 800 mV generates a maximum electric field of 25 mV/mm. The applied voltage has been monitored during the experiment using an oscilloscope and the settings at the voltage source have been adjusted for every experiment to ensure the delivery of 800 mV. The time component of ES regimes varied as a subject of study: the frequency has been set at 1 and 50 Hz and the pulse width deliver duty cycles (DC) of 0.05%, 2.5% and 25% (see Table 1).

**Table 1:** Electrical Stimulation Regimes.

Regime	Amplitude (mV)	Frequency (Hz)	Pulse width	Duty cycle (%)
ES1	800	1	500 µs	0.05
ES2	800	50	500 µs	2.5
ES3	800	1	250 ms	25

The study of the ES regimes has been taken further by describing our stimulation chamber as an electric circuit, Fig. 1A. In this equivalent circuit, the electrode/electrolyte interface is interpreted as a constant phase element (CPE), representing the capacitance of the electric double layer (C_dl_), in parallel with a resistor that accounts for the transfer of charge associated with chemical (faradaic) reactions (R_f_)^32,33,43^. The values of the components of the equivalent circuit are extracted from electrochemical impedance spectroscopy (EIS) at biologically relevant conditions, Fig. 1B. By fitting the EIS Nyquist plots, the values of CPE, R_f_ and the resistance of the cell culture medium (R_e_) are obtained. C_dl_ is extracted from the CPE parameters using the method described previously^33,43^. These values are: C_dl_ = 64.3 μF, R_f_ = 7210 Ω, and R_e_ = 173 Ω (see Fig. 1B).

This equivalent circuit allows the calculation of the voltage delivered to the cell culture medium (V_e_) as well as the current flowing through it (I_e_). We have obtained I_e_ and V_e_ using LT-Spice and plotted them in Fig. 1C. These simulations have been compared and are in reasonable agreement with experimental chronoamperometry results (Supplementary Fig. S2), indicating that this equivalent circuit is a good approximation to describe our system. I_e_ corresponds to the charge moving in the system per unit of time. Therefore, we define the injected charge (Q_In_) as the integral of I_e_ within the length of the applied pulse. When the voltage goes back to zero the C_dl_ discharges; the integral of I_e_ within the discharging period is the recovered charge (Q_Out_). The balance between Q_In_ and Q_Out_ is the total accumulated charge (Q_Acc_) in the system (Q_Acc_ = Q_In_−Q_Out_).

Charges injected into the system are of two types: capacitive charges, associated with ionic flow, and faradaic charges, associated with chemical reactions. The contribution of each type of charge can be extracted from the equivalent circuit. Upon the application of voltage to this circuit, most of the current contributes to build the double layer (i.e., charge C_dl_). As C_dl_ is being charged, I_e_ decreases. Our simulation results show that in regimes ES1 (1 Hz, DC = 0.05%) and ES2 (50 Hz, DC = 2.5%), the attenuation of I_e_ during the applied pulse is negligible due to the short pulse width in these regimes (500 μs). Thus, Q_In_ = 2.2 μC/pulse is small and mostly capacitive in these regimes (see Supplementary Fig. S3). In ES3 (1 Hz, DC = 25%), where long voltage pulses of 250 ms are applied, the contributions of both C_dl_ and R_f_ are significant. Once C_dl_ is fully charged (within ~ 30 ms), I_e_ decreases dramatically, but it stays above zero, meaning that charge continues to be injected via faradaic channel R_f_ (see the inset in Fig. 1C). In this case, Q_In_ = 38.5 μC/pulse with capacitive contribution of ~ 26 μC/pulse (see Supplementary Fig. S3 for details). Q_Out_ is purely capacitive for the three regimes. Figure 1D shows the value of Q_Acc_ in the system after one hour of stimulation for each regime. Results show that Q_Acc_ for ES3 is 49.2 mC/h, while in ES2 and ES1 Q_Acc_ is 6.4 and 0.1 mC/h, respectively, which is proportional to the faradaic contribution.

The electric field present across the cell culture medium (E = V_e_/d) is not proportional to the applied field due to the dynamics of charge and discharge of C_dl_. We define a term denoted as *electric field effective time* (EF “ON”), which is equal to the time span in which V_e_ remains above V_app_/*e* (800 mV/*e* ≈ 300 mV), Fig. 1E. In the case of ES1 and ES2, the pulse width is so short that the attenuation of V_e_ is negligible. Therefore, EF “ON” per pulse is equal to the pulse width (500 μs). Thus, the larger the frequency, the larger the effective electric field time (1.8 s for ES1 vs. 90 s for ES2). In the case of ES3, where the pulse width is long (250 ms), C_dl_ saturates in the first ~ 30 ms and results in the fast drop of V_e_. Therefore, for ES3, EF “ON” per pulse is ~ 5 ms. Moreover, due to the large V_e_ undershoot during discharge, a reverse electric field is also built and collectively gives rise to an alternating effective electric field time of 37.8 s.

### Neuronal morphology is enhanced by increased accumulated charge

**Fig. 2:**
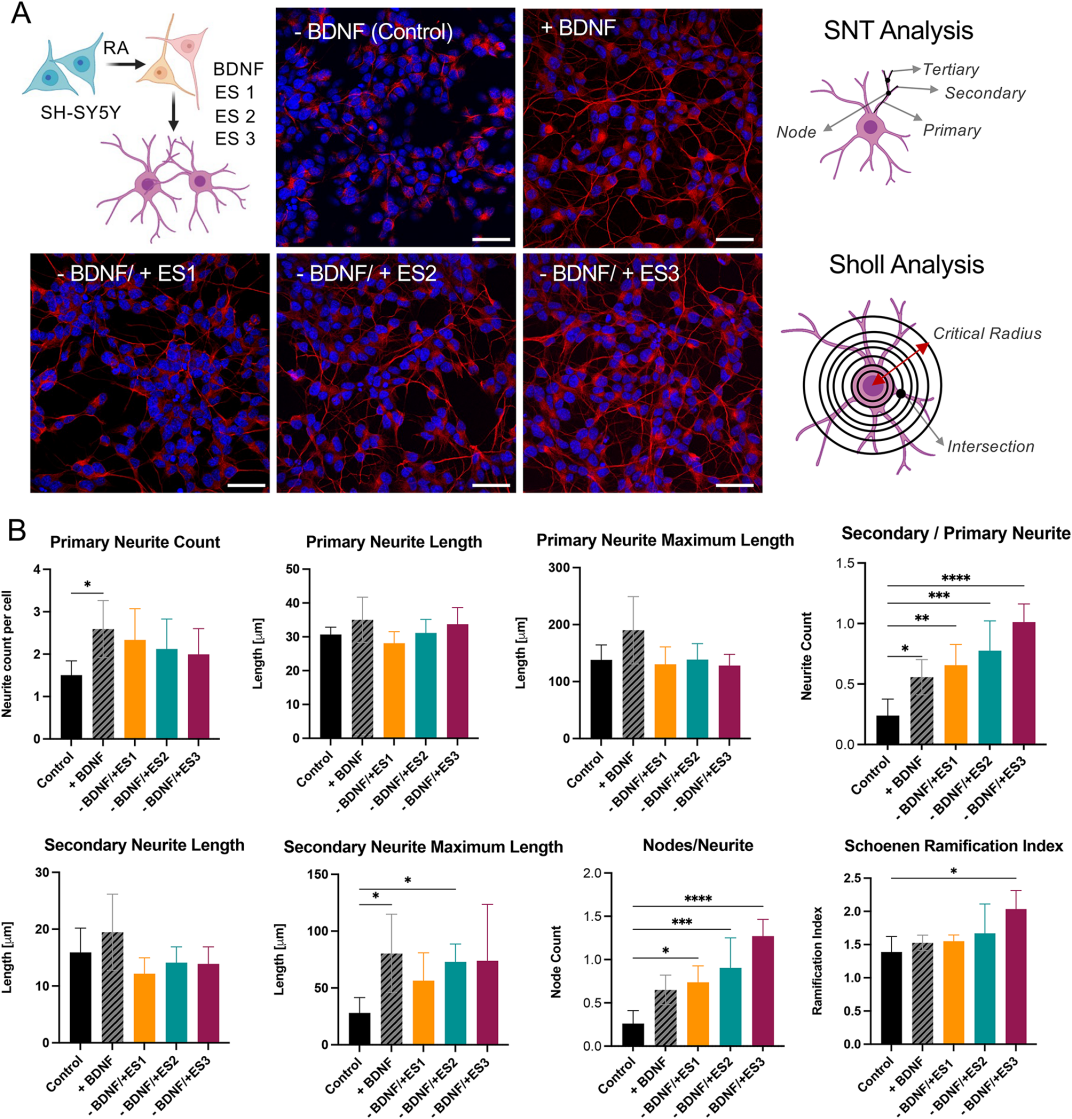
Neuronal Morphology. (**A**) Schematic of experimental design and confocal microscopy images of SH-SY5Y cells, DAPI (blue) and β-tubulin (red). Experimental groups include: no treatment (− BDNF (Control)), BDNF treatment (+ BDNF), − BDNF/ES treatment with three regimes: ES1, ES2 and ES3; scale bar = 20 μm (left). Schematics describing the main parameters quantified by SNT (top) and Sholl (bottom) analysis (right). (**B**) Primary and secondary neurite counts, neurite length, maximum length, number of nodes, intersections, and Schoene ramification index calculated from Sholl analysis. Graphs represent mean values ± standard deviation. Statistical analysis was carried out by one-way ANOVA (*p < 0.05; **p < 0.01; ***p < 0.005; ****p < 0.001).

We analysed the effect of ES on the neuronal morphology of the human neuroblastoma cell line SH-SY5Y and compared it to the effect of BDNF. SH-SY5Y has been treated with RA for 5 days to stop proliferation and initiate differentiation. Then, cells were exposed to ES with regimes described above (− BDNF/ES), treated with BDNF (+ BDNF), or kept untreated (Control), for 3 days, Fig. 2A. Cell morphology was visualised via immunofluorescence (IF) staining of β-tubulin and the neurite outgrowth, branching were quantified. In addition, Sholl analysis^44^ was performed and several relevant morphological indicators, including Schoene ramification index (SRI)^45^, were extracted.

Primary, secondary, and tertiary neurites were manually traced across at least three biologically independent experiments, using six random images from different regions of the well for each experiment. The tracings were measured using the built-in Simple Neurite Tracer (SNT) software within the Neuroanatomy plugin of ImageJ^46^, Fig. 2B. We calculated the number of neurites, their lengths, and the intersections (nodes) where the neurites branched. While the length of primary neurites did not differ among the groups, the number of primary neurites was slightly higher in the electrically stimulated groups and in BDNF treated cells compared to the control (no treatment). Cells treated with BDNF exhibited more primary neurites per cell. Interestingly, neurite branching, as indicated by the number of nodes, and the number of secondary neurites were both significantly higher in cells with ES treatment compared to the control. While all ES regimes positively influenced the acquisition of neuronal morphology compared to the control, there are meaningful differences between them. Among the treated groups, ES3 demonstrated the most extensive arborization. These results indicate that the increase in the number of secondary branches and nodes correlates with accumulated charge.

We also analysed the sophistication of neuronal network using SRI derived from Sholl analysis. SRI is defined as the ratio between the maximum number of intersections and the number of primary branches (see detailed methodology in Supplementary Data S4). The SRI provides information on the degree of neurite arborization. Our analysis confirmed that the SRI is higher for cells treated with ES and BDNF compared to untreated cells (Control). Interestingly, cells exposed to a greater accumulated charge through ES3 treatment demonstrated a significantly higher SRI, indicating a more complex network and increased branching.

Overall, we observed that SH-SY5Y treated with ES acquire a neuronal-like morphology compared to those cells that underwent no treatment. As the accumulated charge associated with ES regimes increases, the complexity of the neuronal network improves. In the case of ES3, which introduces maximum Q_Acc_, the arborization and node counts exceed those obtained with BDNF treatment.

### Synaptic and neuronal markers expression increase with accumulated charge

**Fig. 3:**
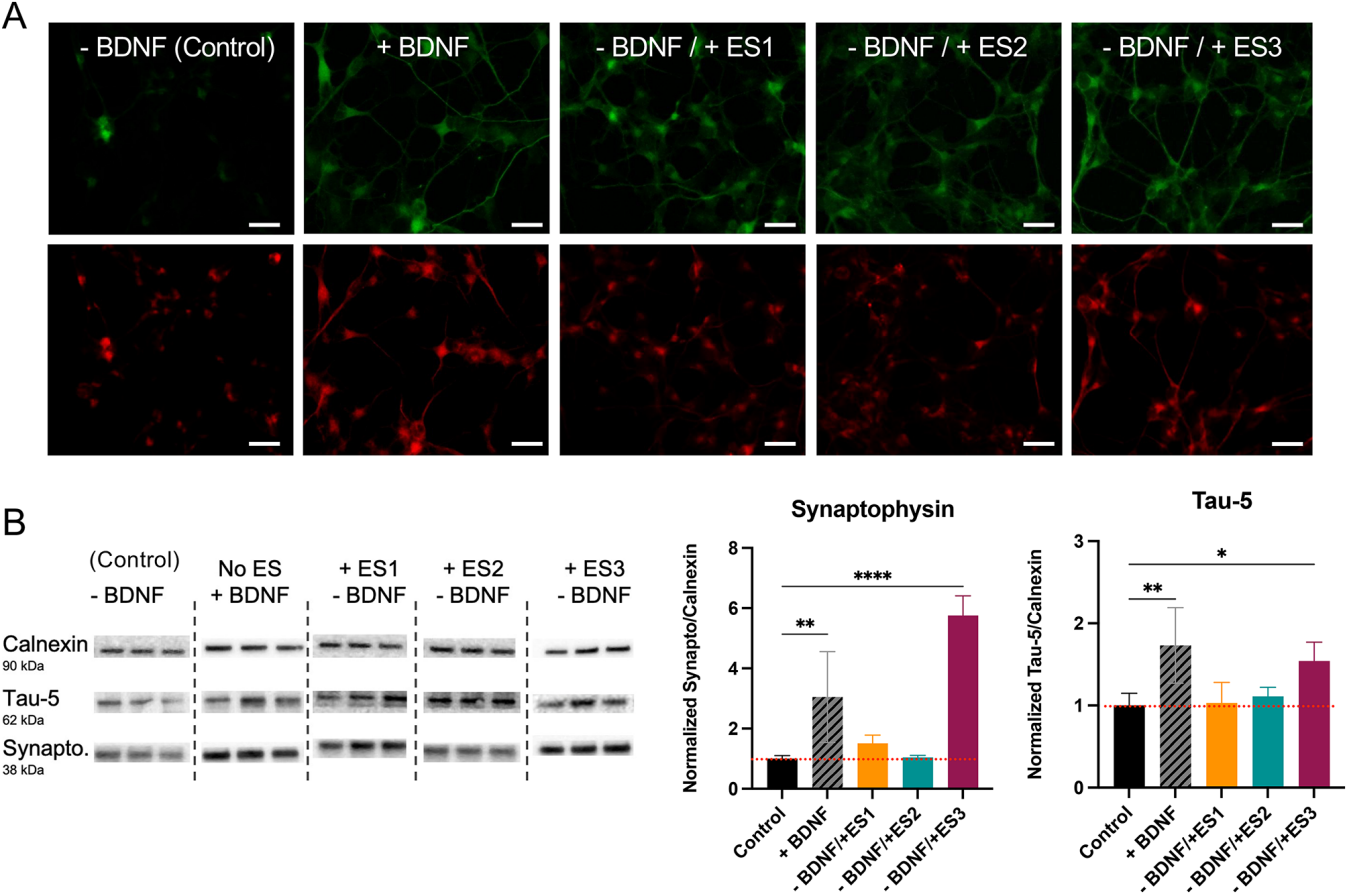
Neuronal and synaptic markers. (**A**) SH-SY5Y cells stained with axonal marker Tau-5 (green), and dendritic marker MAP-2 (red); Scale bar = 20 μm. (**B**) Expressions of Tau-5 and Synaptophysin quantified by means of western blot and normalized against Calnexin. Graphs represent mean values ± standard deviation. Statistical analysis was carried out by one-way ANOVA (*p < 0.05; **p < 0.01; ****p < 0.0001).

The expression of dendritic and axonal markers, MAP-2 and Tau-5, was studied using IF, demonstrating that both markers were present in cells treated with either BDNF or ES, as shown in Fig. 3A. IF images show that the expression of MAP-2 and Tau-5 increased in all groups that received treatment compared to the control. The expression of Tau-5 quantified by western blot (Fig. 3B) shows a significant increase in BDNF and ES3 treated cells compared to ES1, ES2 and no treatment. This data agrees with the trend observed in the quantified length of primary neurites, Fig. 2B. To explore the neuronal maturity and function, we measured the expression of synaptophysin as a presynaptic marker. The expression of synaptophysin is remarkably increased in cells treated with BDNF and ES3. ES3 specifically showed more than a sixfold increase in the expression of this marker, indicating a higher potential for synapse formation in these cells compared to those treated with BDNF, Fig. 3B.

Overall, the expressions of axonal and synaptic markers are increased in ES3 and BDNF treated groups compared to control, but not in ES1 and ES2. Similar to the morphological analysis results, ES3 with larger Q_Acc_ gives rise to more sophisticated neuronal network, a hallmark for neuronal differentiation and maturation.

### Electrical stimulation affects the metabolic activity and the cell cycle

**Fig. 4:**
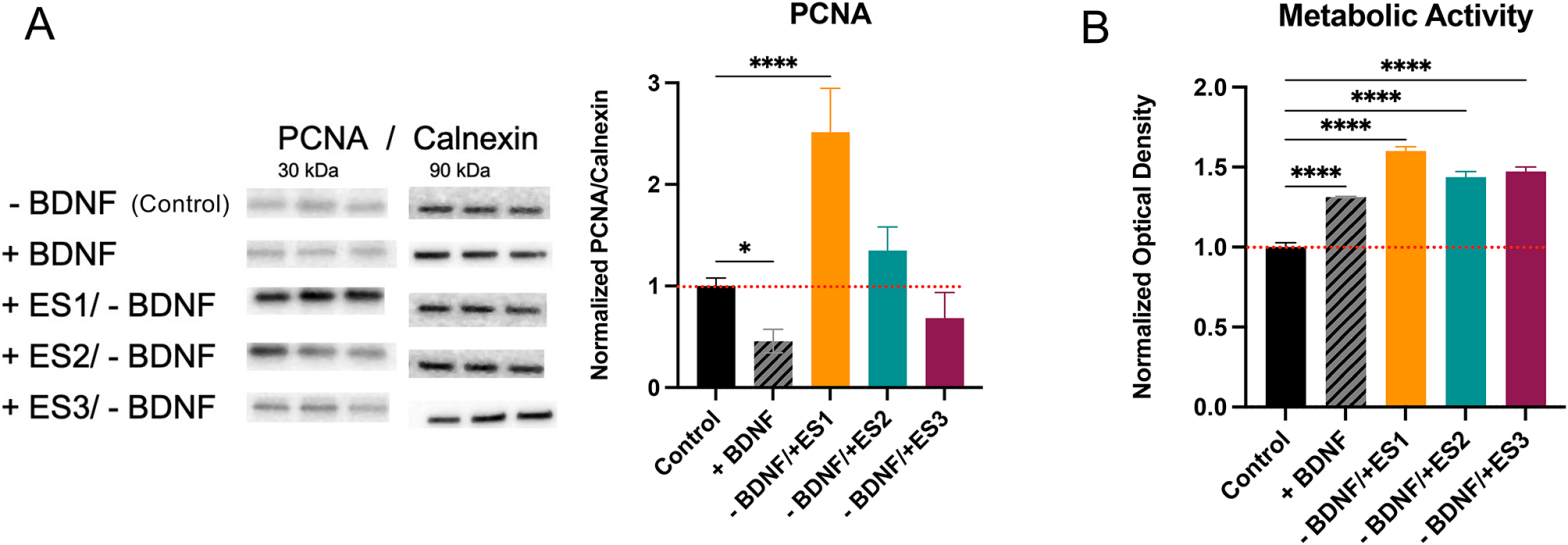
Proliferation and metabolic activity. (**A**) Expression of proliferating cell nuclear antigen (PCNA) quantified by western blot. (**B**) Metabolic activity quantified by alamar blue assay the day after last stimulation. Graphs represent mean values ± standard deviation. Statistical analysis was carried out by one-way ANOVA (*p < 0.05; ****p < 0.0001).

Cell cycle acceleration and proliferation have been frequently reported in several cell lines stimulated with low frequency electrical pulses^47–49^. We quantified the expression of cyclin, also known as proliferating cell nuclear antigen (PCNA), by western blot as a marker for cell proliferation and DNA replication, Fig. 4A.

As anticipated, treatments with BDNF and ES3, both of which significantly promote neuronal differentiation and maturation, show reduced PCNA expression compared to the control (no treatment), with the reduction being statistically significant for BDNF. However, this is not the case for cells treated with the ES1 regime. The significant increase in cyclin expression, peaking during the S phase, suggests a notable change in the cell cycle in response to low frequency/low duty cycle short pulses of ES1. PCNA expression increases slightly with ES2, but this change is not significant.

We assessed the metabolic activity, Fig. 4B, of the cells the day after the final ES treatment (harvest day). All treated cells exhibited increased metabolic activity compared to the control. This increase may partly result from enhanced neurite branching, outgrowth, and possibly synaptic activity, which recruits more mitochondria to the spines and projections of the cells, leading to higher metabolic activity^50,51^. Specifically, for ES1, the elevated metabolic activity could be additionally linked to the accelerated cell cycle.

### Electrical stimulation modulates the extracellular vesicles secreted from SH-SY5Y

Extracellular vesicles (EV) are increasingly recognized as key mediators of cell-to-cell communication and multifunctional signalling units, delivering a wide range of effectors to recipient cells that influence critical cellular processes^52,53^. In neuronal differentiation and maturation context, EV are crucial for regulating synaptic plasticity and promoting both processes^54–57^. Moreover, they are recently studied for their contribution as a type of neurotransmitter^58^. Observing that electrical stimulation promotes differentiation and maturation of SH-SY5Y, we found it relevant to analyse the impact of ES on the characteristics of EV secreted from these cells.

**Fig. 5:**
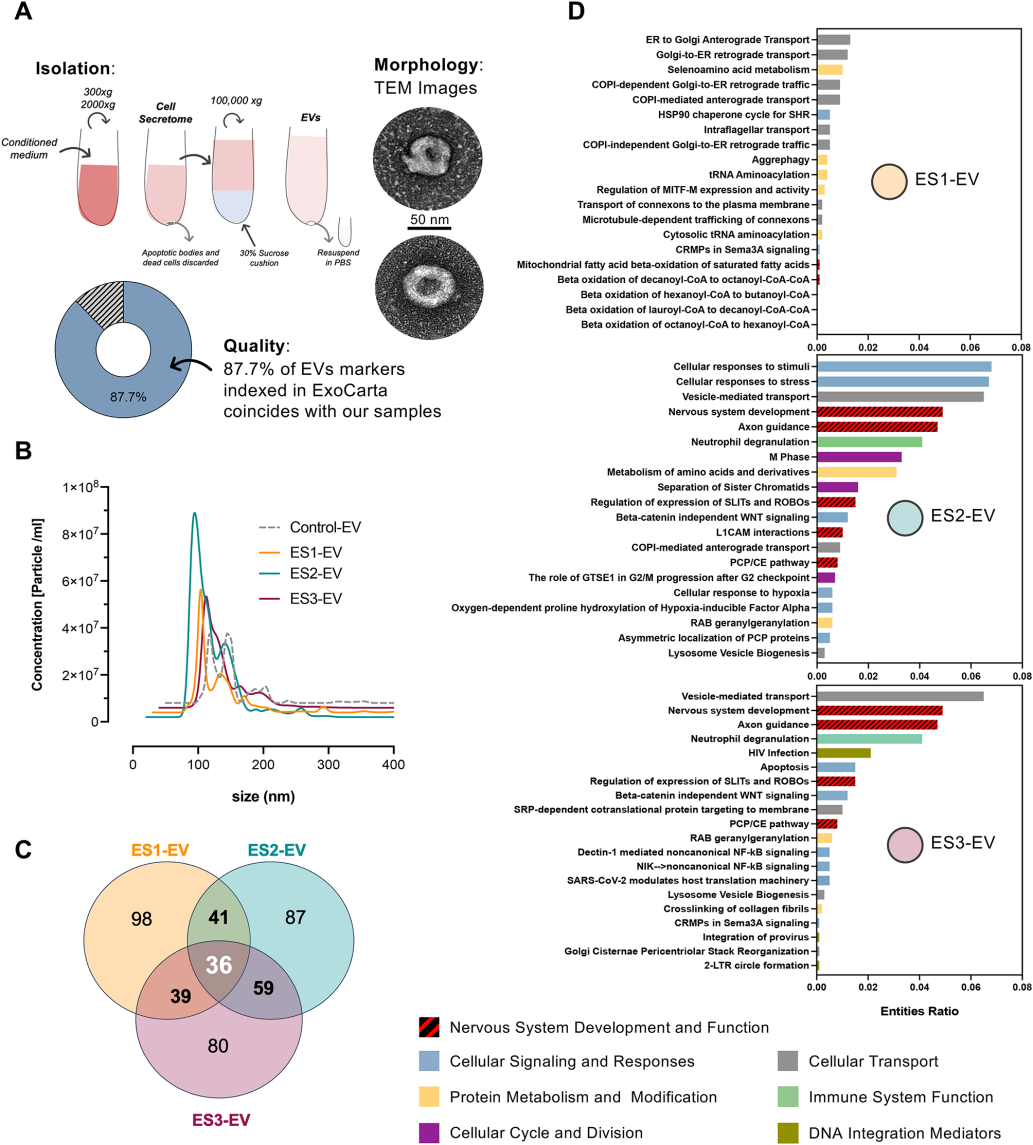
Analysis of extracellular vesicles (EV) secreted from electrically stimulated SH-SY5Y. (**A**) EV isolation and purification method using density gradient and ultracentrifugation at 100 k×*g* ^59^. Transmission electron microscopy (TEM) images of EV obtained from control (top) and electrically stimulated cells (bottom). Pie chart of EV typical markers. (**B**) Concentration and size distribution of EV obtained from electrically stimulated (ES1-EV, ES2-EV, and ES3-EV) and no ES (Control-EV) cells measured by Nanoparticle Tracking Analysis (NTA). (**C**) Venn diagram demonstrating the number of significantly abundant unique proteins found ES1-EV, ES2-EV, ES-3EV versus Control-EV. (**D**) EV cargo proteome analysis, obtained by Reactome, displaying enriched protein groups and pathways in ES1-EV, ES2-EV, ES-3EV versus Control-EV (larger images of each graph are available in the Supplementary Fig. S6).

Results show that the ES treatments and the isolation method used^59^ did not adversely affect SH-SY5Y EV morphology (roundness and membrane integrity), as evidenced by transmission electron microscopy (TEM) images, Fig. 5A. Additionally, quality control analysis revealed that 87.7% of EV markers found in these vesicles were listed in ExoCarta^60^. This indicates that EV obtained in this experiment are of high quality and rich in exosome population, Fig. 5A (see Supplementary Data S5 for the full list of the exosomal markers found in our samples). The concentration and size distribution of EV collected from ~ 2.5 M cells under ES treatments (ES1-EV, ES2-EV and ES3-EV) or no treatment (Control-EV) have been measured by Nanoparticle Tracking Analysis (NTA), Fig. 5B. The mean size (~ 107 nm) and size distribution of the particles demonstrate that EV extracted under all conditions are consistently similar in size, falling within the expected ranges for exosomes (50–150 nm) and small microvesicles (200–1000 nm). This indicates that none of the ES treatments had an adverse effect on physical characteristics of EV. Moreover, the average concentration of EV extracted from electrically stimulated cells is 1.5–2 times higher than that of the control. This suggests that ES enhances EV secretion, with higher frequencies and longer time of EF “ON” further benefiting the process.

The proteomics results showed that several proteins are expressed only under ES regimes compared to the control. Among these, 36 proteins are common across all ES regimes and at least 80 proteins are unique to each specific condition, Fig. 5C. A deeper analysis of these proteins for each regime reveals that the enriched pathways and protein families are also specific, Fig. 5D. ES1-EV shows the lowest enrichment in terms of entity ratios compared to ES2-EV and ES3-EV. Moreover, the pathways enriched in ES1-EV are predominantly linked to cellular transport and protein metabolism. In contrast, ES2-EV and ES3-EV exhibit a high expression of proteins associated with nervous system development and function. This is in agreement with the greater degree of differentiation observed in these groups compared to the control and ES1 treated cells.

Overall, every ES regime imprints a distinct protein signature on cargo and concentration of the secreted EV in SH-SY5Y. Interestingly, ES regimes with larger charge accumulation and longer time for EF “ON” modify EV cargo toward enriched pathways related to nervous system development and function.

## Discussion

In this work, electrical stimulation (ES) has been introduced as an effective tool to mature neuronal cells and express sophisticated network, providing a viable alternative to chemical interventions in vitro. We have demonstrated that pulsed ES induces effects on neuronal differentiation and maturation that are similar to, or even more pronounced than, those achieved with exogenous neurotrophic factors. Furthermore, we have shown that specific ES parameters control distinct cellular responses.

Traditionally, the effectiveness of ES has been challenged by two primary factors: first, the fundamental mechanisms of *electrotransduction* are not yet fully understood, and second, the specific ES parameters that trigger relevant cellular responses remain unclear. We focused on the latter, as the lack of standardisation in ES protocols is the major challenge in mechanism studies. While previous studies provide evidence that ES benefits neuronal function and differentiation (see comprehensive review^61^), the rationale of ES parameters used in these studies cannot be judged. Moreover, the existence of correspondence between ES parameters and biological responses has not yet been established. Therefore, the approach to optimise ES method remains unclear.

Due to the aqueous nature of biological environment, the presence of electrochemical phenomena under direct ES cannot be disregarded^32^. In direct stimulation, the amplitude of ES is limited by the water-splitting voltage and the generation of faradaic by-products. Thus, increasing the strength of ES do not necessarily enhance desirable cellular responses. In fact, high amplitude ES can be detrimental for cells due to critical changes in the chemistry of the extracellular environment^62^. Besides, an electrical double layer forms at the electrode/electrolyte interface, functioning as a capacitor. This causes the current delivered to the system to be frequency dependent. Frequency has been hypothesized to play an important role in triggering cellular responses^61–64^. However, it remains unclear whether the primary factor is the time components of ES (frequency and pulse width), or the amount of charge injected and accumulated in the system. These time components also determine the duration of the effective electric field experienced by the cells. Hence, the question is: how to design relevant ES parameters to study and control biological responses?

In this study, we analysed the roles of accumulated charge (Q_Acc_) and duration of effective electric field (EF “ON”) as measurable physical effectors. These parameters can be modified in the system by adjusting the time components of ES regimes. The strength of ES has been fixed within the safe voltage window (800 mV anodic voltage pulses) and three ES regimes are designed based on these novel criteria: ES1 with Q_Acc_ = 0.1 mC/h and EF “ON” = 1.8 s; ES2 with Q_Acc_ = 6.4 mC/h and EF “ON” = 90 s; and ES3 with 49 mC/h and EF ON = 37.8 s. It is challenging to find equivalent regimes in the literature, since in the majority of in vitro studies, ES regimes were designed and described based on applied parameters at the voltage or current source. The configuration and properties of the electrodes, which are essential for determining the equivalent circuit and ensuring accurate interpretation of the delivered signal^33^, are often not reported. As a result, the electrochemical properties of the reported ES devices remain largely unknown, making it difficult to reproduce the ES conditions in other studies. The criteria introduced in this work aim to bypass differences between the devices, focusing on measurable physical effectors: Q_Acc_ and EF “ON”.

In our experiment, SH-SY5Y cells in all groups were treated with RA prior to the application of BDNF or ES, facilitating the expression of TrkB, the specific BDNF receptor. When exogenous BDNF is introduced (+ BDNF group), the TrkB-BDNF interaction triggers pathways that lead to neuronal differentiation and maturation as previously reported^12,65^. Our results show that cells treated with BDNF express significant neurite outgrowth as well as arborization and pre-synaptic marker, synaptophysin, compared to the control group, which received only RA. This is in agreement with several other reports demonstrating the mutual effect of RA and BDNF in neuronal differentiation and maturation^66–68^.

In the case of ES treatment (− BDNF/ES), particularly for ES3, and to some extent for ES2, we observed a similar response to that seen in + BDNF group. Interestingly, the larger charge accumulation resulted in abundant neural branching, larger SRI, and higher expression of presynaptic marker compared to BDNF treatment. This implies that electrical stimulation with large enough Q_Acc_ prevails over chemical stimulation with BDNF. Accompanying the neuronal maturation response to ES3 and ES2, the cargos of EV secreted from cells subjected to these stimulation conditions are largely enriched in pathways related to nervous system development and axonal guidance. Moreover, SLIT proteins and their receptors (ROBOs), which are involved in guiding the migration of neuronal cells and forming neural networks, are also abundantly found. The possible mechanism could be explained through the increase of Ca^2+^ influx and the release of Ca^2+^ from cytoplasmic reservoirs as well-known responses promoted in direct ES. This mechanism has been confirmed previously in several settings, ES regimes, and cell lines^21,69–73^. The elevation of intracellular Ca^2+^ concentration ([Ca^2+^]_i_) leads to the activation of CaMKII and boosts CREB-based BDNF transcription, therefore promoting neural differentiation and maturation. The link between ES, [Ca^2+^]_i_ elevation and neuronal differentiation has been corroborated by Yan et al*.* in dorsal root ganglion neurons^74^. It can be inferred that larger Q_Acc_ leads to higher [Ca^2+^]_i_, a hypothesis we plan to test systematically in future studies. Moreover, as the oscillation frequency of [Ca^2+^]_i_ has been postulated to dictate certain cellular responses^75,76^, the effect of ES frequency beyond Q_Acc_ will be included in our next path of experimentation.

Regarding the overall influence of the analysed ES parameters, Q_Acc_ seems to be the stronger player in the ES effect towards neuronal maturation. Interestingly, the main source of Q_Acc_ is the faradaic channel (see Supplementary Fig. S2). This suggests that controlled levels of chemical reactions within the safe voltage window are beneficial for promoting differentiation. This supports in vivo and clinical reports that confirm more pronounced regenerative effects while using direct ES compared to capacitive and inductive ES^77,78^. Finally, we demonstrated that a longer presence of the electric field can compensate the reduced Q_Acc_ as evidenced by ES2.

When Q_Acc_ is very low (0.1 mC/h), in the case of ES1, the differentiation response is minimal. ES1-EV cargo neither shows the enrichment of proteins associated with nervous system development and function pathways. However, ES1 regime is not without effect, as it promotes the cell proliferation marker PCNA by sixfold compared to the control. Previous studies on several cell lines have reported that ES can amplify proliferation and accelerate the cell cycle^49,79^. Although some studies reviewed by Love et al.^80^ used voltage ranges similar to the one used in this study, determining an exact equivalent regime remains challenging, as discussed earlier. Nevertheless, this observation supports that specific ES parameters give rise to distinct cellular responses.

EV are therapeutically relevant due to their small size and ability to transport neural differentiation signals^81–86^. However, they encounter challenges like low production yields and limited control over their cargo. Our results show that ES significantly enhances the yield of EV secretion, being this effect more pronounced for a longer presence of electric field. Moreover, ES parameters imprint a distinct signature on the protein composition of EV. Boosting the EV production by means of ES has been recently discussed in a couple of studies. Fukuta et al*.* found that continuous ES at 0.3–0.5 mA/cm^2^ increases EV secretion from a murine melanoma cell line^87^. Moreover, Zhang et al*.* demonstrated that ES at 800 mV, with a frequency of 0.5 Hz and DC = 0.25%, increases the yield of EV production from cardiac mesenchymal stem cells and enhances their cardioprotective effects^88^. There are several overlaps between pathways that are activated by ES and those involved in EV biogenesis. However, we could not find a published study that directly investigate the mechanisms of EV secretion under ES. One potential mechanism involves [Ca^2+^]_i_ elevation under ES, which impacts both the plasma membrane and the cytosolic machinery responsible for protein synthesis and EV biogenesis^89^.

## Conclusions

The benefits of using ES as a non-chemical method for neural differentiation and maturation are numerous. This includes the development of in vitro models free from treatments with neurotrophic factors for drug discovery and research. We show that pulsed voltage-controlled ES with sufficient Q_Acc_ (~ 50 mC/h) is an effective alternative to BDNF for maturing neuronal cells. The well-defined ES method used in this study proved to be more effective than chemical interventions in generating sophisticated neuronal networks and promoting the expression of synaptophysin. Additionally, we demonstrate that the cellular response to ES is tuneable and it is controllable by adjusting ES parameters. Finally, when cells are stimulated with large enough Q_Acc_ and sufficient EF “ON, their secreted exosomes are significantly enriched with neural development associated proteins. This is promising for the future development of electro-engineered therapeutic exosomes.

We described a reproducible electrochemical characterisation and modelling approach to bypass the variability between ES devices and methods reported in previous studies. We introduced novel criteria for defining and designing ES regimes that are applicable across various in vitro ES systems and can be expanded to in vivo and clinical settings. By dissecting Q_Acc_ and EF “ON” as measurable physical effectors, we showed a controllable and meaningful correlation between these ES parameters and unique cellular responses. Our future work focuses on elucidating the regulation of the most hypothesized ES secondary messenger, [Ca^2+^]_i_, and its oscillation, in relation to Q_Acc_, EF “ON”, and ES frequency.

## Methodology

### SH-SY5Y culture and pre-differentiation

Human neuroblastoma cell line SH-SY5Y was obtained from ATCC (American Type Culture Collection) and expanded in 100 mm culture treated plates (Thermo Fisher Scientific) using growing medium (Composition: DMEM + 10% FBS + 1% Antibiotics); all ingredients either purchased from Thermo Fisher Scientific or Gibco. Cells were maintained in a humid incubator at 37 °C and 5% CO_2_. For pre-differentiation of SH-SY5Y, cells were seeded into tissue culture treated 8-well plates (Nunc) at the density of 3 × 10^4^/cm^2^ in growing medium. For immunofluorescent study, cells are seeded on glass coverslips (Sigma), coated with 1% rat tail collagen (Gibco) in sterile phosphate-buffered saline (PBS) to increase cell adhesion. The day after seeding, cells were washed with warm PBS to remove the excess FBS and exposed to pre-differentiation medium (Composition: Neurobasal (Gibco) + B27 (Gibco) + 10 μM Retinoic Acid (Sigma Aldrich) + GlutaMAX (Gibco)) for 5–6 days; medium has been changed every 2–3 days.

### Experiment timeline and study groups

After 5–6 days of treatment with the pre-differentiation medium, the cells were divided into the groups described in Table 2 and treated for 3 days with either Blank medium (Composition: Neurobasal + B27 + GlutaMAX) as the Control, or BDNF medium (Neurobasal + B27 + GlutaMAX + 50 ng/mL BDNF (BioNova Científica S.L.)), or Blank medium + electrical stimulation treatments (ES1, ES2, and ES3). Cells were harvested the next day. We conducted at least 3 independent experiments, each consisting of three 8-well plates (24 individual wells) for each condition.

**Table 2:** Experimental groups.

Group	Medium	Electrical stimulation	Post assessment
− BDNF (Control)	Blank medium	NO ES	IF, WB, EV
+ BDNF	BDNF medium	NO ES	IF, WB
− BDNF/+ ES1	Blank medium	ES regime 1	IF, WB, EV
− BDNF/+ ES2	Blank medium	ES regime 2	IF, WB, EV
− BDNF/+ ES3	Blank medium	ES regime 3	IF, WB, EV

### Electrical simulation (ES)

All ES treatments were applied for 1 h per day, over 3 consecutive days, aiming to perform the treatments at the same time each day. Table 1 summarizes the parameters used in each ES regime. ES was applied to the cells using a custom-made setup^38^, Fig. 1A. Briefly, the stimulator chamber consisted of an 8-well culture plate lid attached to a printed circuit board (PCB), where pure platinum wire electrodes were secured. These electrodes were soldered in parallel to deliver the same voltage to each well. The PCB was connected to a signal generator (Agilent 33220A, 20 MHz Function/Arbitrary Waveform Generator) with fixed series output impedance of 50 ohms. Since the output voltage value depends on the impedance of the system connected to the generator, we monitored the delivered output with an oscilloscope (Rohde & Schwarz RTB2004 Digital Oscilloscope, 2.5 GSa/s) and adjust the input parameters for having the desired V_well_. The electrodes were cleaned and sterilized using sonication in pure water for 3 min, followed by 70% ethanol, and were exposed to UV light for at least 20 min inside a laminar flow hood before each experiment.

### Electrical impedance spectroscopy (EIS)

Electrochemical characterisations were performed using a potentiostat with FRA32M module (PGSTAT 204, Autolab, Metrohm Hispania) in a three-electrode setup. An Ag/AgCl electrode, with 6 mm diameter (RE-1B, BioLogic) was used as a reference electrode. Working electrode and Counter electrode were assigned arbitrarily to any of the electrodes of the stimulation device, as they are equivalent. The distance between the electrodes was fixed at 32 mm. 4 mL fresh DMEM w/o phenol red (Gibco) was loaded to the system as electrolyte for each measurement. The EIS has been carried at biological conditions inside the incubator at 37 °C and 5% CO_2_. EIS is performed in the frequency window of 10 mHz to 50 kHz with a sinusoidal excitation signal of 10 mV, with DC applied voltage of 0 V. Bode and Nyquist diagrams were obtained and analysed in Nova 2.1 software. EIS measurements have been done after 50 cycles of cyclic voltammetry (CV), aiming to clean the surface of the electrodes from impurities that influences the obtained results. The open circuit potential (OCP) for each measurement was recorded to be reproducible with small standard deviation being ~ 0.2 V.

### Cyclic voltammetry (CV)

CV tests have been performed using the same setup described above, within the voltage window (− 0.6 V to 0.9 V), where no reactions associated with water hydrolysis have been observed. Several cycles were performed with a sweep rate of 0.1 V/s and a step of − 10 mV until the system became stable before final measurement. Moreover, in order to determine the safe window, additional CV measurements have been carried out, increasing the voltage window by 0.5 V at each limit until we reach the interval (− 1.1 V to 1.4 V). 5 cycles were performed at each range, with a sweep rate of 0.1 V/s and a step of − 1 mV.

### Metabolic activity assay

Metabolic activity was measured using alamarBlue™ reagent (Thermofisher Scientific). After incubating live cells with the working reagent at the concentration recommended by the provider for 2.5 h, media were transferred to a 96-well plate in triplicates and the absorbance was measured at 570 nm, with a reference wavelength of 600 nm. The optical density measured directly correlates with the cell metabolic activity.

### Immunofluorescence assay (IF)

To prepare the samples for IF, cells were initially cultured on standard borosilicate glass coverslips (diameter: 18 or 25 mm; thickness: 0.17 mm). On the day of harvest, cells were fixed with 3.8% paraformaldehyde (PFA) in filtered phosphate-buffered saline (PBS), with the pH adjusted to 7.4. Cells were permeabilized using 0.1% Triton X-100, in filtered PBS and the non-specific binding was blocked using 3% bovine serum albumin in filtered PBS. Cells were incubated with primary antibodies for 2 h and secondary antibodies for 1 h at room temperature. After a final wash, coverslips were mounted on glass slides using Mowiol® and kept at 4 °C.

For morphological analysis, we used mouse β-tubulin (Cat No. 322600, Thermo Fisher Scientific) at a dilution of 1:1000, and the secondary antibody anti-mouse Alexa Fluor® 488 (Thermo Fisher Scientific) at a dilution of 1:1000. DAPI (Invitrogen) was used at a dilution of 1:5000. Cells were imaged using confocal microscope spinning disk SpinSR10 coupled to an inverted microscope IX83 (Olympus), using a 60X/1.3 silicone Super Apochromat (SAPO) objective.

For neural and axonal marker expression analysis, we used rabbit anti-MAP2 polyclonal antibody (Cat No. PA517646, Thermo Fisher Scientific) at a dilution of 1:1000, and mouse Tau monoclonal antibody (TAU-5) (Cat No. AHB0042, Thermo Fisher Scientific) at a dilution of 1:1000. Secondary antibodies included anti-rabbit Alexa Fluor® 488 and Alexa Fluor® 546, both from Thermo Fisher Scientific. Cells were imaged using an Olympus BX51 fluorescence microscope, equipped with a Hamamatsu ORCA camera, using 20X/0.45 objective.

### Image analysis

FIJI (ImageJ) software with the Neuroanatomy plug-in was used to analyse neurite morphology^46^. Briefly, IF images were subjected to contrast adjustment, background removal, and LUT inversion to enhance the clarity of the neuronal structures. Neurites were then carefully selected, traced, labelled, and measured using the Simple Neurite Tracer (SNT) tool within the Neuroanatomy plug-in. The number of neurites, neurite length, and the number of nodes per neurite were calculated for each condition. For Sholl analysis^44^, the NeuronJ plug-in was employed to perform bulk analysis on 60–150 cells per image. For each cell, the Sholl profile was generated, calculating the number of intersections, the radius, and the logarithm of the number of intersections divided by the unit area. Additionally, key parameters including the maximum intersection number, maximum intersection radius, ramification index, and branching index were calculated per cell and per experimental condition (Supplementary Data S3).

### Western blot (WB)

Cells were lysed using 5 µL/cm^2^ lysis buffer (Composition: RIPA Lysis Buffer (Thermo Fisher Scientific) + protease inhibitor, Mini Protease Inhibitor Cocktail (Roche) + Phosphatase Inhibitor Cocktail 2 (Sigma)). Every 3 wells from the same experiment and same group combined to generate sufficient amount of protein and working volume. Yet 6 replicates of combined samples generated and at least 4 replicates were tested for statistical relevance. Protein concentrations were determined using BCA Assay (Thermo Fisher Scientific), and 20 μg of protein was loaded in Mini-PROTEAN TGX stain-free precast PAGE gels (4–15%) (BioRAD). Following electrophoresis, proteins were transferred to nitrocellulose membranes using semi-dry transfer method. Membranes were blocked for 1 h at room temperature then incubated overnight at 4 °C with primary antibody. After washing with TBST, the membranes were incubated with relevant HRP-conjugated secondary antibodies. Protein bands were visualized using ECL chemiluminescence (Thermo Fisher Scientific) reagent and captured with UVP BioSpectrum 810 Imaging System and bands intensities were quantified using Image J. Each marker was visualised before the next antibody were applied. Primary antibodies used for WB include: Mouse Tau monoclonal antibody (TAU-5) (Cat No. AHB0042) at the dilution of 1:1000; rabbit anti-Calnexin endoplasmic reticulum marker (Cat No. ab22595) at the dilution of 1:10,000; rabbit anti-synaptophysin antibody, YE269 (Cat No. ab32127) at the dilution of 1:6000; and mouse anti-PCNA monoclonal antibody (Cat No. D54474) at the dilution of 1:20,000.

### Extracellular vesicles (EV) extraction, purification, and characterisation

EV were isolated from cell culture media and characterised following ISEV guidelines^90^. Briefly, the collected medium from each study group was centrifuged at 300×*g* for 20 min at 4 °C to remove cell debris, followed by a second centrifugation at 2000×*g* for 20 min at 4 °C to eliminate apoptotic bodies and large vesicles. 4 mL of 30% sucrose (Merk) in filtered PBS at 4 °C was loaded in 38.5 mL, open-top ultracentrifugation tube (CAT. 344058, Beckman). The supernatant obtained previously (cell secretome) was loaded to the tube slowly without disturbing the sucrose cushion and subjected to ultracentrifugation (Beckman XP-100 Ultracentrifuge with a swinging bucket rotor) at 100,000×*g* at 4 °C for 2 h^59,91^. 32 mL of the supernatant was removed and replace with filtered PBS to apply extra wash and subjected to additional ultracentrifugation at 100,000×*g* at 4 °C, for 1.5 h. The pellet was collected and resuspended in 50 μL filtered PBS and stored at − 80 °C before characterisation. EV were characterised by nanoparticle tracking analysis (NTA) device (Malvern Panalytical NanoSight NS300 Instrument). Samples were diluted 100 times in milli-Q water, the camera sensitivity was set at 12–14, and 5 individual captures were taken for each sample at room temperature. The concentration and particle size reports were generated using NanoSight software.

### EV proteomics

EV were lysed in 5% SDS with protease and phosphatase inhibitors, followed by cysteine reduction and alkylation. Proteins were then digested using trypsin on S-trap columns, and the resulting peptide pool was purified and desalted using Oligo R3 polymeric reverse phase. Peptides were quantified using a QuBit fluorometer. Tryptic peptides were separated via nano HPLC and analysed using the Orbitrap Exploris 240 mass spectrometer, with label-free relative quantification applied between experimental conditions. Peptide and protein identification were performed using Proteome Discoverer v 2.5, incorporating Mascot, Sequest HT, and MSFragger search engines with a target-decoy strategy to ensure FDR < 1%. Identified proteins underwent label-free quantification, with data normalized and statistical significance determined using t-tests and Benjamini–Hochberg correction. Abundance ratios were expressed as log2 for symmetrical up/down regulation analysis.

### Pathway enrichment analysis

The proteomic data obtained from SH-SY5Y-derived EV were analysed using the REACTOME bioinformatic tool (an open-source, manually curated, peer-reviewed pathway database)^92^ to identify pathways enriched in ES-EV vs. Control-EV. The protein list subjected to the analysis were selected from the abundance and significant list (FDR < 1%), provided from proteomics analysis. Protein list further filtered for those that are at least pronounced in 2 biological replicates. Enriched pathways were quantified by calculating the *entities ratio*, defined as the number of entities in a specific pathway relative to the total number of entities for the entire species (Homo sapiens) within the selected molecular type. These pathways were categorized by related functions based on literature and the results were visualized using GraphPad.

## Supplementary Information

Supplementary Information.

## Data Availability

Data is provided within the manuscript, supplementary files, and are available on open access Digital CSIC repository (https://digital.csic.es/).

## Abbreviations

BDNF: Brain-derived neurotrophic factor
C_dl_: Capacitance of the electric double layer
CPE: Constant phase element
CV: Cycle voltammetry
DMEM: Dulbecco’s modified eagle medium
ECL: Enhanced chemiluminescence
EF “ON”: Electric field effective time
EIS: Electrochemical impedance spectroscopy
ES: Electrical stimulation
EV: Extracellular vesicles
FDR: False discovery rate
IF: Immunofluorescence
NTA: Nanoparticle tracking analysis
PBS: Phosphate-buffered saline
PCNA: Proliferating cell nuclear antigen
PFA: Paraformaldehyde
PI3/Akt: Phosphoinositide 3-kinase/protein kinase B pathway
Q_Acc_: Accumulated charge
ROBO: Roundabout receptor
RAS/MAPK: Ras/mitogen-activated protein kinase
SDS: Sodium dodecyl sulphate
SH-SY5Y: A human-derived neuroblastoma cell line
SLIT: Slit guidance ligand
SNT: Simple neurite tracer
TrKB: Tyrosine kinase receptor B
WB: Western blot
