# Supplementary material for: Bioelectric stimulation outperforms brain derived neurotrophic factor in promoting neuronal maturation
Source: Sci Rep. 2025 Feb 8;15:4772. doi: 10.1038/s41598-025-89330-4 (PMC11807145; doi:10.1038/s41598-025-89330-4)

Supplementary Information

Bioelectric Stimulation Outperforms Brain Derived Neurotrophic Factor in Promoting Neuronal Maturation

María del Pilar Diego-Santiago[1], María Ujué González[1], Esther María Zamora Sánchez[1], Nuria Cortes-Carrillo[1], Carlos Dotti[2], Francesc Xavier Guix Ràfols[3], Sahba Mobini*[1]*

[1]*Instituto de Micro y Nanotecnología, IMN-CNM, CSIC (CEI UAM+CSIC), Isaac Newton 8, 28760 Madrid, Spain*

[2]*Departamento de Biología Molecular and Centro de Biología Molecular "Severo Ochoa" (UAM-CSIC), Universidad Autónoma de Madrid, 28049 Madrid, Spain*

[3]*Department of Bioengineering, Institut Químic de Sarrià (IQS), Universitat Ramón Llull (URL), 08017 Barcelona, Spain*

*Corresponding Authors:

Sahba Mobini sahba.mobini@csic.es

Francesc Xavier Guix Ràfols francesc.guix@iqs.url.edu

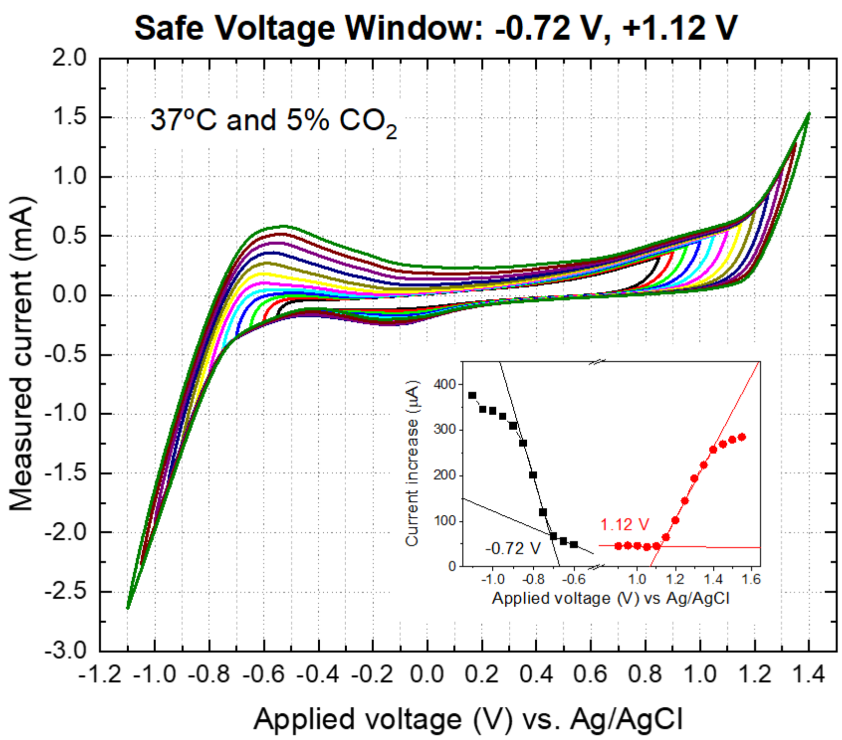

Figure S1. Determination of the safe voltage window for Pt electrodes. We performed consecutive cyclic voltammograms, increasing the voltage range by 0.5 V at each limit until we reach the interval (-1.1 V – 1.4 V). We plotted the increase of the current at the two extremes between each cycle and obtained S-shape graphs (see the inset). The first dramatic change in the current increase at a given voltage implies the outbreak of a new charge injection mechanism, which in this case corresponds to water splitting. We determine these turning point voltages through the intersection of the linear fits of each region.

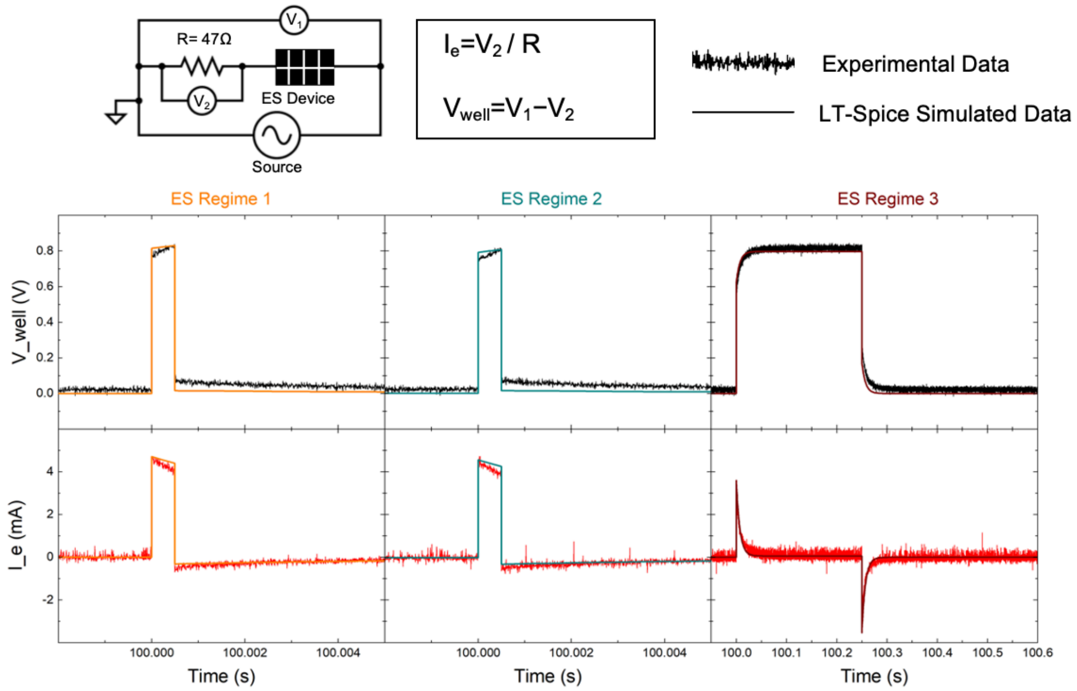

Figure S2. Experimental Pulse Amperometry. To experimentally measure the delivered current, we have replicated the electrical stimulation conditions (regimes) using the same

source used for ES experiments and placed an additional known resistor (R= 47 Ω) between the source and the ES device, as shown in the circuit above. Using an oscilloscope, we measured V_1 and V_2, and calculated the delivered current ($I_e=V_2/R$) during time. The data were collected from the oscilloscope and plotted (red plot). The actual V_{well} data were also collected and plotted ($V_{well} = V_2 - V_1$) (black plot), showing a very close approximation to the ideal pulses used as input for LT-Spice simulation. Despite being slightly noisy, the experimental results closely match the LT-Spice simulations (depicted with solid lines coloured according to each regime). The slight mismatch is attributed to the deviation between the actual impedance of the system and the equivalent circuit values calculated from EIS.

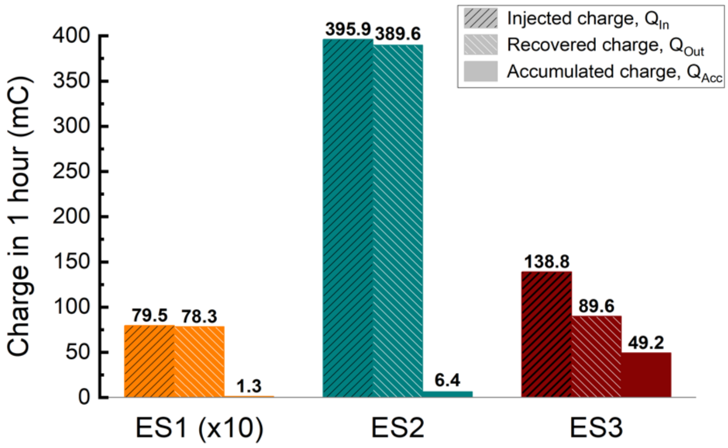

Figure S3. The total charge injected (Q_{in}), recovered (Q_{Out}) and accumulated (Q_{Acc}) in the system during one hour of stimulation for each regime. The maximum capacitive charge contribution to Q_{in} can be obtained from the value of C_{dl} and the applied voltage ($Q_{Capacitive} = C_{dl} \times V_{app}$). For ES3, where C_{dl} fully charges, the capacitive charge is equal to this maximum value (= 25.7 μC/pulse). The rest of the contribution to Q_{in} is faradaic ($Q_{faradaic} = V_{app}$ / (14.6 kΩ × pulse width) = 13.7μC/pulse). For ES1 and ES2, Q_{in} is mainly capacitive because the pulse is so short that during this interval C_{dl} is short circuit ($Q_{Capacitive}$ = Pulse width × V_{app} / 173Ω (R_e) = 2.3 μC/pulse). The faradaic contribution for these regimes is only 0.027 μC/pulse. Q_{Out} is mostly purely capacitive in all cases and therefore the Q_{Acc} is mainly due to $Q_{faradaic}$ of Q_{in}. Q_{Acc} is larger in ES2 compared to ES1 because of the higher frequency.

Data S4. Extensive methodology for Sholl analysis and calculation of relevant morphological indicators. Neuron morphology analysis presents a challenge due to the lack of standardized protocols capable of systematically comparing neurons with varied morphologies and complexities. To address this, we employed Sholl Analysis, a technique first introduced by D.A. Sholl in the 1950s, which has remained a fundamental method for quantifying neuron arborization. The analysis involves drawing concentric circles around the neuron's soma and counting the intersections of dendrites with these circles. From these counts, we generated Sholl Intersection Profiles (SIPs), allowing us to calculate key morphological parameters. We applied Sholl Analysis to SH-SY5Y cells using the NeuronJ software. NeuronJ facilitates high-throughput, batch processing of neurons, enabling us to analyse 60 to 150 cells per image. Pre-traced neurons were subjected to Sholl Bulk Analysis, which generated .csv files for each cell, containing metrics such as the number of intersections per circle, radius, and various calculated statistics (e.g., soma position, start and end radii, maximum intersection number, and mean intersections). Additionally, we derived parameters

such as the Ramification Index and Branching Index to quantify neurite complexity. The SIP data was fitted to a semi-logarithmic function,

$$\log\left(\frac{N}{S}\right) = -k\,r + m,$$

where N represents the number of intersections, S is the normalizing area ($S = \pi \cdot r^2$), k is the Sholl Decay Parameter, and m represents the y-intercept. The fit provided the Sholl Decay Coefficient and the regression coefficient R^2. We used this approach to extract key metrics for each experimental condition, including Control, + BDNF, ES1, ES2, and ES3 treatments. We focused on the following parameters: Max. intersection number, Max. intersection radius, Ramification Index, Branching Index, and Sholl Decay Coefficient. For each condition, we computed the mean and standard deviation across multiple images (at least 4 biological replicates). This approach allowed us to compare neuron morphology across different experimental treatments.

Study Group	Average max. number of Intersections	Critical Radius	Sum Intersec.	Mean Intersec.	Ramification Index	Branching Index
**Control (AVE)**	2.87	14.77	63.32	1.77	1.39	22.89
**Control (SD)**	0.26	4.00	3.88	0.17	0.23	7.76
**+ BDNF (AVE)**	4.46	18.16	104.75	2.27	1.53	73.73
**+ BDNF (SD)**	0.67	3.25	27.13	0.22	0.12	36.15
**ES1 (AVE)**	4.37	15.63	93.44	2.18	1.55	79.99
**ES1 (SD)**	0.60	1.56	18.02	0.27	0.09	26.32
**ES2 (AVE)**	15.68	86.50	1.93	1.67	49.15	3.81
**ES2 (SD)**	1.84	19.43	0.36	0.44	19.62	1.21
**ES3 (AVE)**	4.30	17.94	94.43	2.03	2.08	53.55
**ES3 (SD)**	1.15	4.92	20.63	0.28	0.61	23.47

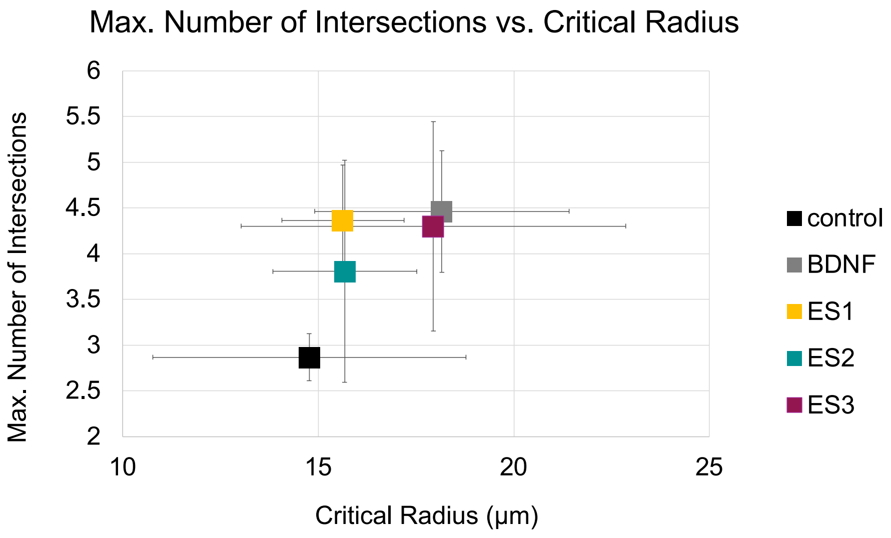

Data S5. List of exosomal markers in isolated EV that overlap with ExoCarta:

CBX3, Itgam, AMY1A, Mfge8, ME1, Hspa8, RAB1B, Actb, VIM, Gnai2, STAU1, Anxa2, GOT1, Cd9, SSB, Hsp90ab1, SUPT16H, CD63, XPNPEP1, DPP4, PODXL, EPS8L3, SYPL1, BAIAP2L1, SLC39A14, MUC13, NOP58, PTGFRN, PLD3, LDHA, GLRX, PGK1, RAB13, GAPDH, ALAD, HLA-A, RCC2, ENO1, HINT1, HSP90AA1, RAP2C, CKB, EIF2S3, PKM, TMED9, EPCAM, ILF2, DPEP1, SPARC, DPP4, ACTG1, KRT9, ALCAM, STX3, TPM4, STXBP2, PLCB4, MEP1A, CLEC3B, GPA33, NCKAP1, Cd86, PSMC6, Cd40, CTSC, Cd40lg, TUBB2B, Itgb2, RAB5A, Icam1, UBE2D3, Anpep, CP, Rps6ka1, CEP55, Anxa6, PSAT1, Cdc25c, SCPEP1, Actg1, NAP1L1, Actb, KRT33B, Cd86, KLK7, Icam1, RAB18, Hspd1, KRT19, Hspa8, DPYSL3, Hspa8, MVB12B, Hspa8, HNRNPH3, Hspd1, S100P, Lamp2, TPP1, Cd9, TENM3, Pdcd6ip, LY6D, Itgb2, SNRPD3, Hsp90ab1, VPS35, Tubb5, PRPF19, Anxa7, PRDX5, Tsg101, LGALSL, Gdi2, SERPINB5, Eif4a2, PCOLCE, Anxa1, APOD, Anxa4, ITGA2, Lgals3, CLU, Sdcbp, CAPN1, Ywhah, C11orf54, Ywhaz, PSIP1, Ywhag, RPL30, Prdx1, HAPLN3, Rab11b, PA2G4, Rab7, RTN3, Cfl1, KRT27, H3f3a, TUBA4A, Hist1h2bf, EIF6, Hist1h2bj, CTSV, Hist1h2bl, DSC3, Hist1h2bn, TSPAN14, Hist2h2aa1, DYNLL2, Hist2h2aa2, TXLNA, Hist4h4, HADHB, Pfn1, RPS5, CD81, ITGAV, HLA-B, CDC37, HLA-DRA, ATP2B4, HLA-DRB5, HSPA9, HLA-DRB1, RAB2A, PTPRC, KRT71, GNAI2.

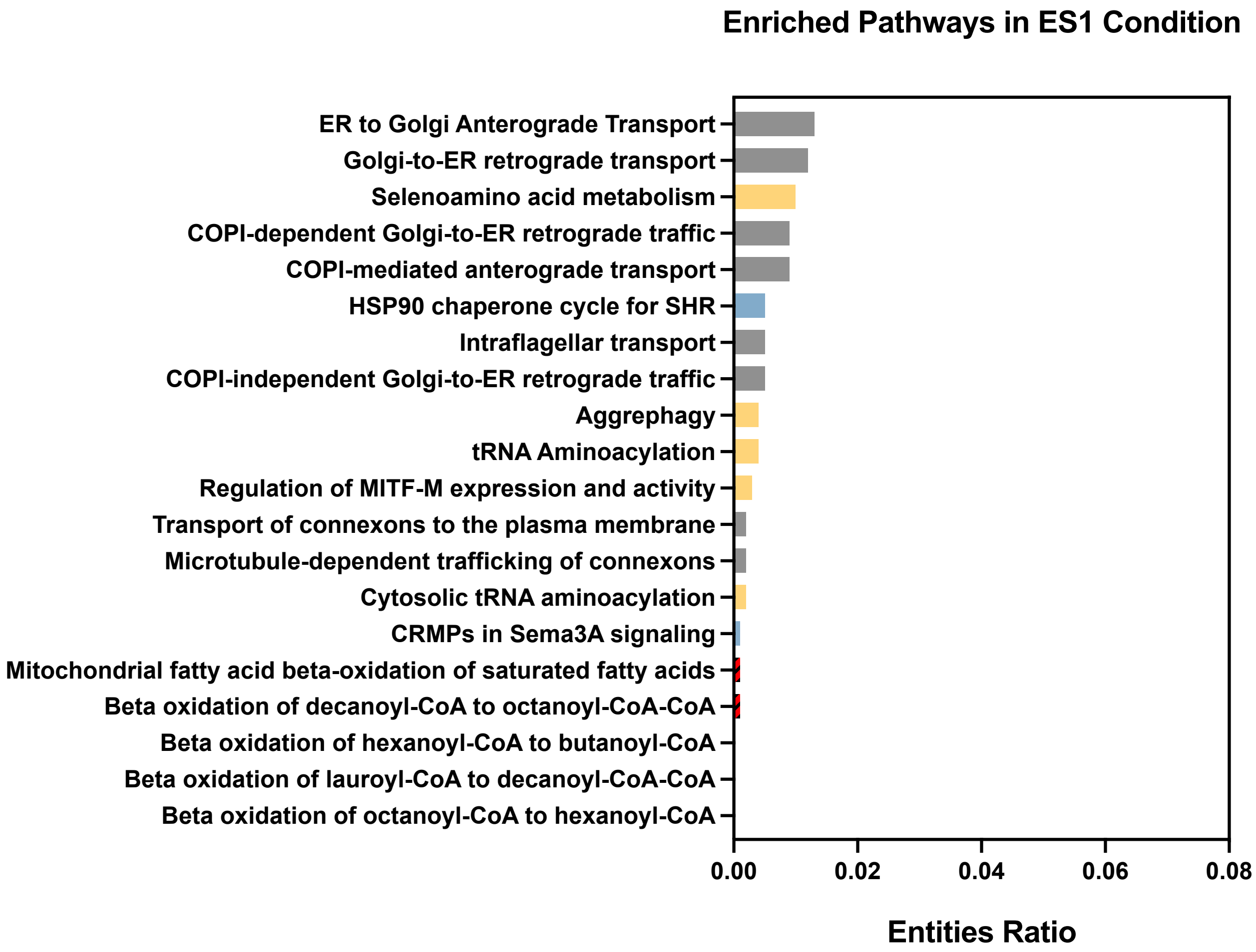

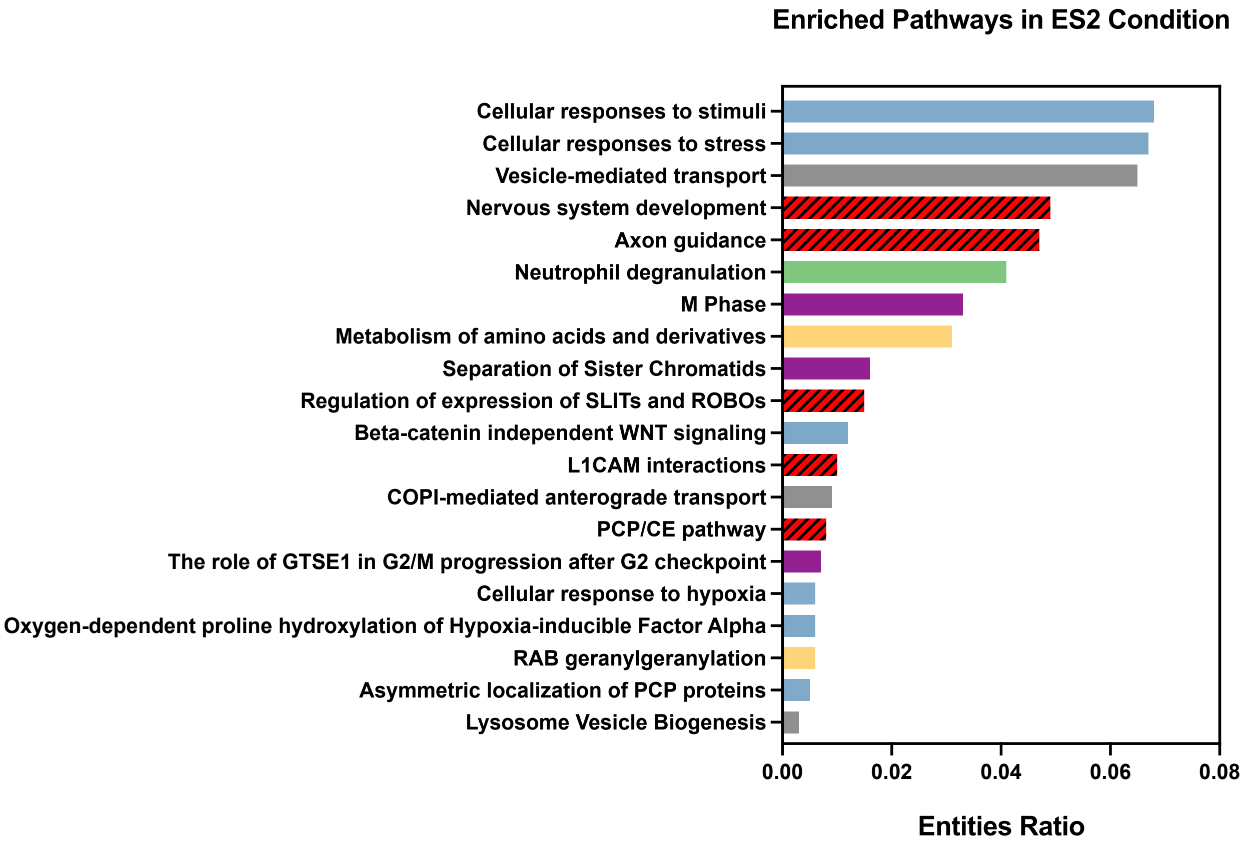

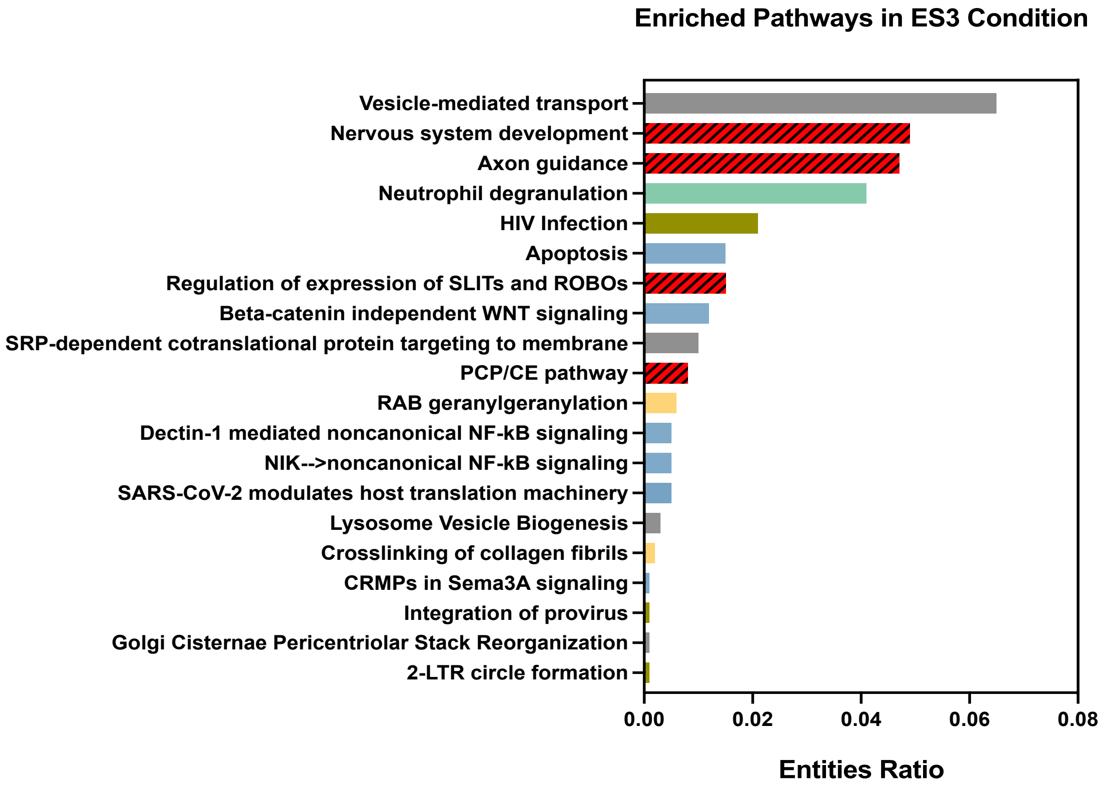

Figure S6. Enriched protein groups and pathways in EV extracted from each ES condition vs. control (Pathway analysis by Reactom).

Original Images of Blots/Gels: Each highlighted square has been selected as representative data, with outlier data excluded from the quantification. When possible, all replicates have been included in the quantification.

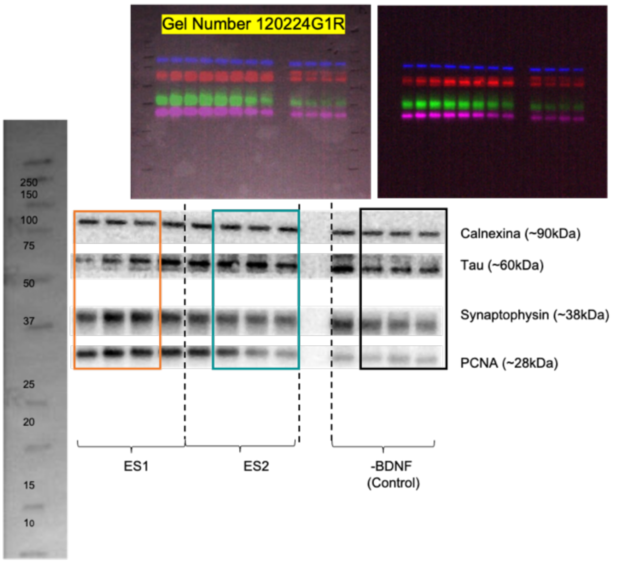

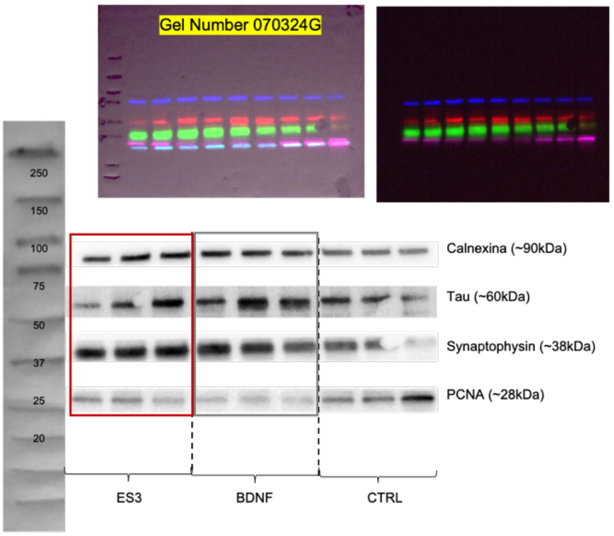

Figure 3

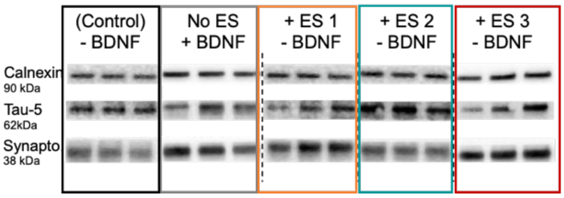

Figure 4

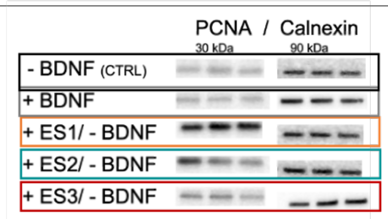

Supplement: Supplementary file 1 — Supplementary Information. [file 41598_2025_89330_MOESM1_ESM.pdf]
